# Pathogenesis of sarcopenia in chronic obstructive pulmonary disease

**DOI:** 10.3389/fphys.2022.850964

**Published:** 2022-07-19

**Authors:** Kai Ma, Fengxiang Huang, Ruiping Qiao, Lijun Miao

**Affiliations:** Department of Respiratory Disease, First Affiliated Hospital of Zhengzhou University, Zhengzhou, China

**Keywords:** COPD, sarcopenia, pathogenic factors, pathogenesis, treatment

## Abstract

Chronic obstructive pulmonary disease (COPD) is a common pulmonary disease characterized by persistent respiratory symptoms and airflow obstruction. In addition to lung diseases, chronic obstructive pulmonary disease (COPD) is often associated with other organ diseases, and sarcopenia is one of the common diseases. In recent years, multiple factors have been proposed to influence muscle dysfunction in COPD patients, including systemic and local inflammation, oxidative stress, hypoxia, hypercapnia, protein synthesis, catabolic imbalance, nutritional changes, disuse, ageing, and the use of medications such as steroids. These factors alone or in combination can lead to a reduction in muscle mass and cross-sectional area, deterioration of muscle bioenergy metabolism, defects in muscle repair and regeneration mechanisms, apoptosis and other anatomical and/or functional pathological changes, resulting in a decrease in the muscle’s ability to work. This article reviews the research progress of possible pathogenesis of sarcopenia in COPD.

## 1 Introduction

Chronic obstructive pulmonary disease (COPD) is a complex, heterogeneous and multicomponent disease that, in addition to abnormal lung function, is often complicated by other organ diseases, such as cardiovascular disease, metabolic disorder, osteoporosis, muscle disease, cachexia-associated anxiety and depression, and gastrointestinal diseases. These complications can independently affect patient mortality and hospitalization rates (Pizarro and Skowasch, 2019).

Sarcopenia is a syndrome characterized by reduced muscle mass, strength and fitness (Cruz-Jentoft et al., 2019). Among the multiple complications of COPD, the prevalence of sarcopenia is 21.6% (Benz et al., 2019), which varies with age, disease severity and IBODE score (BMI, obstruction, dyspnoea and exercise ability) (Dudgeon and Baracos, 2016). Sarcopenia can affect both respiratory and nonrespiratory muscles. Studies have shown that many factors, including systemic inflammation and oxidative stress, hypoxia and hypercapnia, protein synthesis, catabolic imbalances, nutritional changes, smoking, endocrine dysfunction, ageing, and some drugs (e.g., steroids), affect muscle function and have a systematic effect on all muscle groups (Gea et al., 2015; Laveneziana et al., 2019). Individually or in combination, these factors can lead to a reduction in muscle mass and cross-sectional area, deterioration of muscle bioenergy metabolism (factors closely related to fibre type ratio, mitochondrial activity, or the availability of muscle’s own blood flow), deficiencies in muscle repair and regeneration mechanisms, and anatomic and/or functional pathological changes, such as apoptosis, resulting in a decrease in the muscle’s ability to work (Whittom et al., 1998; Levine et al., 2003; Pitta et al., 2006). Sarcopenia in patients with COPD can have many negative effects, including reducing the patient’s quality of life, increasing hospitalization and mortality rates, and increasing the medical burden of the society. Therefore, an in-depth understanding of the pathogenic factors and pathogenesis of this process to find treatments for patients with COPD complicated with sarcopenia is of great importance to improve the quality of life and prognosis of patients. This paper will review the research progress on COPD complicated with sarcopenia in the following ways.

## 2 Pathogenesis of sarcopenia associated with chronic obstructive pulmonary disease

### 2.1 Inflammation

Inflammation is the main feature of chronic obstructive pulmonary disease. Usually, the inflammatory response of COPD patients is not limited to the lungs but is also accompanied by systemic chronic inflammation. Systemic chronic inflammatory responses can lead to sarcopenia in COPD patients. TNFα and IL-6 levels were significantly increased and negatively correlated with grip strength (HGS) skeletal muscle mass index (SMMI) in COPD patients with muscle loss compared with those without muscle loss (Byun et al., 2017).

Various studies have shown that catabolism/anabolism imbalance triggered by systemic inflammation is a major factor leading to skeletal muscle atrophy and decreased muscle strength. First, inflammation increases the amino acids required by the liver for the synthesis of acute phase proteins and reduces the storage of muscle proteins, redirecting amino acids from the muscle to the liver (Engelen et al., 2000). Second, inflammatory factors such as TNF-α activate the ubiquitin-dependent proteolysis system, thereby increasing muscle protein degradation (Llovera et al., 1997). The ubiquitin–proteasome system (UPS) relies on the proteolytic pathway, which is the main pathway of myofibrillar decomposition. The ubiquitin-protein ligase E3 connects the ubiquitin chain to the substrate protein, and the ubiquitinated protein is then recognized and degraded by the 26S proteasome. Ligase E3, including Atrogin-1, MURF-1 and NEDD4, is thought to be a rate-limiting factor in this pathway. Third, elevated plasma proinflammatory cytokine levels have also been reported to reduce anabolic factors by altering the bioavailability and biological effects of key hormones, such as testosterone, insulin growth factor-1 (IGF-1), and dehydroepiandrosterone (DHEA), required for skeletal muscle growth and maintenance. Studies have shown that compared with healthy subjects, the systemic levels of testosterone, IGF-1, and DHEA in COPD patients are significantly reduced and negatively correlated with serum IL-6 levels, and the IL-6/DHEA ratio was found to increase gradually with the exacerbation of peripheral muscle atrophy (Debigare et al., 2003). In addition, inflammation can also be involved in skeletal muscle dysfunction by inducing oxidative stress and apoptosis of muscle cells.

TNF-α is the main inflammatory factor affecting muscular atrophy and cachexia. On the one hand, TNF-α activates the NF-κB signalling pathway, which transfers NF-κB from the cytoplasm to the nucleus, promotes activation of the ubiquitin–proteasome system in myocytes, and increases myofibrillar protein degradation (Li et al., 2008). In response to TNF-α, the IkB kinase complex phosphorylates IkB, leading to ubiquitination and proteasome degradation. This results in the activation of NF-κB, which rapidly enters the nucleus to initiate the transcription factor FOXO and upregulate MuRF1 expression. On the other hand, TNF-α can also inhibit the expression of MyoD and other muscle differentiation growth factors through the NF-κB pathway, resulting in disruption of skeletal muscle differentiation (Reid and Li, 2001). In addition, TNF-α stimulates apoptosis by causing DNA disruption and/or interaction with TNF-α receptors on muscle cells (Kuwano and Hara, 2000). Finally, studies have shown that TNF-α can also directly inhibit myofilaments, independent of changes in protein degradation or synthesis (Reid et al., 2002). Il-6 is another inflammatory factor that causes sarcopenia. It promotes the expression of myostatin and caspase-3 in muscle fibres and increases the hydrolysis of muscle proteins. In addition, gene transcription in the ubiquitin proteasome system is regulated by interaction (activation) with FOXO and NF-κB.

### 2.2 Oxidative stress

Oxidative stress is one of the main driving mechanisms of the occurrence and development of COPD. Oxygen deprivation, chronic inflammation, cigarette smoke, waste and increased respiratory rates all contribute to increased oxidative stress in the lungs. Superoxide anion (O_2_−) and nitric oxide anion (NO−) are the most important free radicals in living organisms. Reactive oxygen species/nitrogen (RON) induces lipid peroxidation of membrane phospholipids, resulting in severe changes in membrane structure and functional properties. In addition, the oxidation or nitration of proteins by RONs can change the structure or chemistry of proteins, resulting in a decrease in protein function. Finally, RONs can interact with DNA to cause DNA damage and deletions, mutations, and DNA-protein crosslinking. Needle biopsy confirmed that the levels of two membrane lipid peroxidation products (4-hydroxy-2-nonenal and lipofuscin) and protein tyrosine nitrification in the lateral femoral muscle cells of patients with moderate to severe COPD were significantly higher than those of healthy subjects at rest (Barreiro, Gea, Corominas, Hussain; Allaire et al., 2002). This suggests that peripheral skeletal muscle in COPD patients is exposed to repeated oxidative stress. Oxidative stress can lead to sarcopenia in the following ways:

#### 2.2.1 Increased skeletal muscle protein degradation

ROS further activate the downstream ubiquitin proteasome system by activating NF-κB, P38 mitogen-activated protein kinase (p38MAPK), and FOXO transcription factors in COPD patients (Powers et al., 2010; Liu et al., 2011; Bernardo et al., 2015). Autophagy is a lysosomal-mediated protein degradation pathway in eukaryotic cells. Under basal metabolism or stress, the adaptive activation of autophagy can cause protein degradation and generate amino acids for recycling, which can have an adaptive protective effect on the maintenance of skeletal muscle mass. However, in COPD patients, an abnormal increase in autophagy leads to muscle protein degradation, resulting in muscle atrophy (Kneppers et al., 2017). Autophagy consists of autophagy initiation vesicle nucleation vesicle extension vesicle contraction autophagosome binds with lysosome to form autolysosomes. Autophagy is initiated by the ULK1(Unc-51-like autophagy activating kinase 1)-ATG13-FIP200 (two protein factors including focal adhesion kinase family interacting protein of 200 kDa) complex. This complex is under the control of the AMP-activated protein kinase (AMPK) and complex 1 of mammalian target of rapamycin (mTORC1) pathways. Skeletal muscle protein degradation can be accelerated through activation of AMPK, inhibition of Akt/mTORC1 signalling (to induce the initiation of autophagy) (Powers et al., 2016) and the activation of calpain, caspase-3 and other proteolytic systems, leading to muscle dysfunction.

#### 2.2.2 Damaged mitochondria

The mitochondrial respiratory chain is the main site of ROS generation in cells and the main target of reactive oxygen species. Mitochondrial DNA is highly sensitive to oxidative damage, which is mainly manifested in two aspects. 1) Induction of mitochondria-mediated apoptosis of muscle cells: Excessive increases in ROS and RNS induce the release of caspase-activating proteins such as cytochrome and apoptosis-inducing factors. On the one hand, high levels of ROS and RNS can change the REDOX potential of cells and promote the formation of mitochondrial membrane permeability conversion pores (Pollack and Leeuwenburgh, 2001). On the other hand, the reaction of ROS and RNS with membrane phospholipids can also increase mitochondrial membrane permeability. These changes promote the release of pro-apoptotic factors such as cytochrome from the mitochondria into the cytoplasm. 2) Induced mitochondrial respiratory chain dysfunction: Studies in animal models (Tretter and Adam-Vizi, 2000; Murray et al., 2003) suggest that ROS and RNS may react directly with lipids and/or proteins to significantly alter the activity of mitochondrial respiratory chain complexes (I, II, IV) and reduce the activity of mitochondrial or Krebs circulating oxidases. In addition, increased mitochondrial permeability due to membrane lipid peroxidation can reduce the ion concentration gradient on the mitochondrial intima and increase the uncoupling of oxidative phosphorylation, which leads to respiratory chain dysfunction (Levraut et al., 2003). Finally, oxidative stress interacts with mitochondrial DNA, resulting in impaired gene expression and structure of respiratory chain polypeptides encoded by the mitochondrial genome (Wei et al., 1998), resulting in respiratory chain defects.

#### 2.2.3 Biological function of injured muscle filaments

*In vitro* studies have shown that high intracellular concentrations of ROS and RNS can significantly reduce the activity of Na+/K+ pumps and calcium pumps and plasma pumps, thus affecting membrane depolarization and action potential transmission in cells (Kurella et al., 1995). These modifications may result in a reduced rate of myofilament relaxation, thereby limiting the amount of ATP hydrolysis and the forces generated by actin and myosin interactions, as well as the rate of shortening.

### 2.3 Disuse

COPD patients, with worsening of airflow obstruction, develop hyperinflation and increased dyspnea. Which leads to a decrease in physical activity. Skeletal muscle disuse can lead to several adaptive changes, including a reduction in type I fibre and oxidase capacity, muscle fibre atrophy, and muscle capillary reduction. These changes work together to reduce muscle endurance and strength. The loss of muscle strength and endurance further limits the patient’s mobility, creating a vicious cycle. A comparative study found that type I fibres in the vastus lateralis muscle of healthy sedentary subjects accounted for 41%, while type I fibres in the vastus lateralis muscle of healthy active subjects ranged from 60–65% (Proctor et al., 1995). This suggests that muscle disuse can lead to a one-third reduction in the proportion of type I fibres. However, studies have also shown significant differences in quadriceps muscle endurance between COPD patients and healthy subjects despite similar levels of physical activity (Couillard et al., 2002). This suggests that disuse can only partially explain the severe morphological, metabolic, and functional abnormalities in the surrounding muscles of COPD patients, with the co-involvement of other pathophysiological factors.

Disuse skeletal muscle atrophy occurs as a result of accelerated proteolysis and decreased synthesis. Reduced physical activity in COPD patients can induce transcriptional activation of FOXO and NF-κB by inducing an increase in ROS in skeletal muscle fibres and a decrease in the IGF-1/Akt signalling pathway (Wu et al., 2011), thereby activating the proteolytic system. Insulin-like growth factor-1 (IGF-1) is a positive regulator of muscle growth and can promote the proliferation, differentiation and maturation of skeletal muscle cells. IGF-1 is the main signalling molecule of skeletal muscle protein synthesis. IGF-1 increases skeletal muscle protein synthesis mainly through the PI3K/Akt/mTOR and PI3K/Akt/GSK3β pathways (Yoshida and Delafontaine, 2020). Phosphorylation of AKT stimulates mTOR, and mTORC1 activation mainly regulates the downstream Factors 4E-BP1 and p70S6K. 4E-BP1 binds to elF4E (eukaryotic cell translation promoter) to inhibit protein expression, and 4E-BP1 phosphorylated by mTOR loses its ability to bind to elF4E, thereby promoting mRNA translation. In addition, the PI3K/Akt/mTOR signalling pathway is inhibited during muscle inactivity, leading to decreased protein synthesis (Hyatt et al., 2019).

### 2.4 Hypoxemia

COPD patients often develop chronic hypoxemia due to progressive airflow obstruction and destruction of the alveolar-capillary exchange surface. Chronic hypoxia leads to the impairment of skeletal muscle function through the inflammatory response, oxidative stress, influence on myogenic differentiation, protein synthesis and catabolism, and myofibre type conversion. In chronic hypoxia, the transformation of type Ⅰ and type Ⅱa fibres from oxidative metabolism to type Ⅱb fibres mainly from glycolysis is considered an adaptation of the body to hypoxia. The proportion of type I fibres in peripheral muscles in COPD patients has been reported to be significantly reduced compared with that in nonhypoxic patients (Gosker et al., 2002) and strongly correlated with the resting value of oxygen partial pressure in their arterial blood.

### 2.5 Drugs–Glucocorticoids

Glucocorticoids (GCs) are commonly used in acute exacerbations of COPD due to their anti-inflammatory properties and sometimes in the long-term maintenance of end-stage disease. GC-induced muscle atrophy is characterized by decreased fibre cross-sectional area and decreased myofibrillar protein content. Long-term effects of GC often lead to shrinkage of muscle fibres, and fast muscle and glycolysis muscle (type Ⅱ fibre) are more susceptible to GC than oxidizing muscle (type Ⅰ fibre) (Fournier et al., 2003). GC not only inhibits muscle IGF-I production but also inhibits IGF-I phosphorylation of EIF4E-binding protein 1 (4E-BP1) and ribosomal protein S6 kinase 1 (S6K1), thereby inhibiting protein synthesis (Shah et al., 2000a; Shah et al., 2000b). In addition, GC inhibits the PI3K/Akt pathway. GC accelerates IRS-1 protein degradation and increases the expression of P85 α protein (the regulatory subunit of PI3K), which reduces PI3K activity (Zheng et al., 2010). Other studies have found that GC inhibits mTOR signal transduction in muscle cells by enhancing the expression of REDD1 (Wang et al., 2006), reduces the phosphorylation of 4E-BP1 and S6K1, and ultimately inhibits protein synthesis.

### 2.6 Dystrophy

COPD patients suffer from reduced appetite, age and drug-related anorexia, resulting in insufficient energy intake and increased protein breakdown, which results in skeletal muscle atrophy, reduced exercise tolerance and decreased quality of life. Between 20 and 70% of COPD patients are known to be malnourished (Raguso and Luthy, 2011). Therefore, nutritional status also plays an important role in the development of sarcopenia. When nutrients are deficient, AMPK senses low nutrient and energy state, inactivates mTORC1, and directly phosphorylates ULK1, enhances ULK1 activity, and promotes the formation of ULK1-ATG13-FIP200 complex, thus initiating the formation of autophagosome. Some studies have found that fasting for 48 h can significantly improve the expression of FOXO (Imae et al., 2003) and induce the expression of ubiquitin systems, including ubiquitin ligases (Atrogin1 and MuRF1) and autophagy-related genes (Milan et al., 2015), thereby activating protein degradation.

### 2.7 Others

In COPD, testosterone, vitamin D deficiency, and exposure to cigarette smoke also contribute to sarcopenia through multiple mechanisms. Testosterone is significantly and positively correlated with respiratory parameters such as forced expiratory volume in 1 s (FEV1), forced vital capacity (FVC), the ratio FEV1/FVC and lean body mass index (LMI) (Slim et al., 2020) and can increase muscle mass by activating satellite cells (Morley, 2017). Therefore, testosterone deficiency promotes the development of sarcopenia. Cigarette smoke exposure not only leads to the slow to fast transformation of muscle fibre types and the reduction of overall muscle fibre size but also enhances pulmonary oxidative stress, inhibits the activation of satellite cells and enhances the ubiquitin proteasome system, leading to sarcopenia (Barnes, 2016a; Chan et al., 2020; Li et al., 2021).

## 3 Treatment

### 3.1 Pulmonary rehabilitation training

Pulmonary rehabilitation training can not only improve the muscle function and exercise tolerance of COPD patients but also improve the dyspnoea and quality of life of COPD patients. Pulmonary rehabilitation training includes a variety of training modes, such as endurance/aerobic training (e.g., walking, cycling, etc.), resistance/strength training (e.g., weights and dumbbells, etc.), interval training, and neuromuscular electrical stimulation. Studies have shown improvements in lower extremity muscle strength, endurance, and quality of life in COPD patients after extensive exercise-based interventions (De Brandt et al., 2018; Kerti et al., 2018). Endurance training leads to a decrease in the proportion of type II fibres and an increase in the peripheral muscle fibre cross-sectional area in COPD patients. It also induces a shift in muscle protein content to less glycolysis and more oxidative metabolism to offset muscle dysfunction in COPD patients (Iepsen et al., 2016). The combination of resistance and endurance training increased the expression of Akt and mTOR proteins, as well as the area of type I and II fibres (Kazior et al., 2016), and was shown to lead to greater improvements in muscle strength and quality of life than endurance training alone. Percutaneous electrical muscle stimulation may be used in patients with severe ventilation and/or cardiac limitations who are unfit to perform pulmonary rehabilitation exercise training.

### 3.2 Nutritional supplementation

Malnutrition is common in patients with COPD and can increase hospitalization and mortality rates. Protein and specific nutritional supplements can enhance pulmonary rehabilitation. Clinical studies have shown that taking multiple nutrients (leucine, vitamin D, and omega-3 fatty acids) in conjunction with exercise training can improve inspiratory muscle strength and physical activity levels in additional ways (van de Bool et al., 2017). SCWD recommends protein supplementation for patients with sarcopenia associated with COPD (1.5 g/kg/day) (Bauer et al., 2019). However, due to the heterogeneity of supplements, outcome measures, and pulmonary rehabilitation programs, evidence on the impact of nutritional supplements on improving outcomes during pulmonary rehabilitation in COPD patients remains inadequate and requires further study.

### 3.3 Drug treatment

Based on the common mechanisms of sarcopenia mentioned above, studies in recent years have begun to explore drugs that can interfere with the muscle proteolytic - synthesis pathway, anti-inflammatory, antioxidant stress and other related mechanisms to increase muscle content, improve muscle strength and function. At present, drug therapy mainly includes vitamin D, antioxidants, androgens, selective androgen receptor modulators, growth hormones, and myostatin inhibitors. Studies have shown that these treatments have the potential to increase muscle mass and improve muscle strength and function, reducing the risk of falls.

#### 3.3.1 Vitamin D

Vitamin D increases inspiratory muscle strength and maximal oxygen uptake in COPD patients (Jans sens et al., 2009). The serum 25(OH)D concentration at the end of summer is significantly and positively correlated with muscle strength, which is a positive predictor of muscle strength and a better quality of life (Kokturk et al., 2018). Vitamin D receptor gene polymorphism in COPD affects quadriceps muscle strength (Hopkinson et al., 2008). Vitamin D deficiency exists in COPD patients, and vitamin D supplementation has been shown to increase proximal muscle strength in adults (Stockton et al., 2011), thereby reducing the risk of acute exacerbation or deterioration in patients.

#### 3.3.2 Testosterone

Endogenous total testosterone levels are significantly reduced in COPD patients and are positively correlated with FEV1, FVC, FEV1/FVC ratio, and lean body mass index (Slim et al., 2020). Testosterone therapy can increase muscle mass by increasing muscle IGF-1 protein expression (Ferrando et al., 2002). Recently, testosterone was found to exert anti-inflammatory effects by inhibiting NF-κB signalling (Wang et al., 2021). However, its clinical use is limited by its possible side effects (increased risk of cardiovascular events and skin diseases). Selective androgen receptor modulators (SARMs) have been shown to have similar results to testosterone with fewer side effects in androgen-dependent tissues, but their implementation in clinical practice remains controversial (Fonseca et al., 2020).

#### 3.3.3 Growth hormone and ghrelin

GH mainly stimulates liver synthesis of IGF-1, triggering the igF-1-ArK-MTOR pathway, which enhances protein synthesis and promotes muscle growth (Bia getti and Simo, 2021). Ghrelin, also known as auxin releasing peptide, is an endogenous ligand of growth hormone secretory hormone receptor (GHSR), which not only promotes growth hormone secretion but also reduces fat utilization and increases appetite. Studies have found that ghrelin not only increases serum IGF-1 levels in COPD patients but also improves exercise-tolerant respiratory muscle strength and body weight of patients (Miki et al., 2012).

#### 3.3.4 Myostatin inhibitors

Bimagrumab is a monoclonal antibody that blocks activin type II receptors and blocks the activity of myostatin and other negative skeletal muscle modulators. A phase II study showed that bimagrumab treatment promoted increased skeletal muscle mass and strength and improved mobility in subjects (Rooks et al., 2017). In COPD patients with reduced skeletal muscle mass, intravenous administration of Bimagrumab at 30 mg/kg over 24 weeks can safely increase skeletal muscle mass (Polkey et al., 2019).

#### 3.3.5 β2 receptor agonist

β2 agonists, traditionally used for the treatment of COPD, have been shown to attenuate and/or reverse muscle atrophy (Joassard et al., 2013). The β2 adrenergic receptor agonist salbutamol increased skeletal muscle protein turnover (protein synthesis and breakdown) after resistance exercise, thereby helping to improve skeletal muscle net protein balance after resistance exercise. In addition, Inhaled bronchodilators will reduce dyspnea and improve exercise performance during pulmonar rehabilitation.

#### 3.3.6 Antioxidants

As mentioned above, antioxidant capacity can affect muscle mass and strength through a variety of mechanisms and is closely related to sarcopenia in COPD patients. Treatment to reduce muscle oxidative stress may help improve the occurrence and development of COPD sarcopenia.

##### 3.3.6.1 N-acetylcysteine, erdosteine and carbocisteine

Mucus dissolvers such as N-acetylcysteine, erdosteine and carbocisteine have antioxidant and anti-inflammatory properties. NAC is a strong reducing cysteine acetyl derivative, which is a precursor for the synthesis of glutathione substrate cysteine. Oral NAC will significantly increase plasma glutathione levels in COPD patients (Bridgeman et al., 1994). After NAC treatment, the exercise endurance of the quadriceps femoris and the sense of respiratory distress and muscle fatigue after exercise were all improved (Koechlin et al., 2004). As mercaptan compounds, erdosteine and carbocisteine can also effectively remove free radicals and oxidants, improve the level of mercaptan in cells, and inhibit the activation of NF-κB and the expression of inflammatory genes (Cazzola et al., 2019).

##### 3.3.6.2 Antioxidant mimics

Antioxidant mimics reduce oxidative stress by restoring depleted intracellular antioxidant enzymes such as SOD, catalase and glutathione peroxidase (GPx). Superoxide dismutase mimicry M40419 can inhibit the increased expression of oxidative stress markers and prevent the development of emphysema in animal models (Tuder et al., 2003). Glutathione peroxidase is a se-dependent antioxidant enzyme, and ebselen is a GPx mimic. Studies have shown that ebselen can significantly reduce the expression of cytokines, chemokines and proteases in the alveolar lavage fluid of mice exposed to cigarette smoke and infected with influenza A virus (Oostwoud et al., 2016).

##### 3.3.6.3 Nrf2 activators

Activation of Nrf2 regulates the expression of antioxidant genes and plays a crucial role in preventing oxidative stress-induced damage in cells and tissues. Sulforaphane (SFN) is not only an activator of Nrf2 but also has anti-inflammatory activity. SFN inhibits the production of inflammatory factors in COPD patients by regulating the TLR (Toll-like receptor) pathway (Zeng et al., 2021). Other studies have shown that SFN and SFNAC (sulforaphane n-acetylcysteine (SFNAC), the main metabolite of SFN), inhibit CSE/PM10-induced oxidative stress and inflammation through the ERK, JNK and Nrf2 signalling pathways (Son et al., 2020). Resveratrol is a plant polyphenol existing in grape skins and seeds. Studies have found that resveratrol has a certain therapeutic effect on a rat COPD model, and its mechanism is related to inhibiting oxidative stress and the inflammatory response in rats with chronic obstructive pulmonary disease (Wang et al., 2017).

#### 3.3.7 Anti-inflammation

IKKβ is an important part of the NF-κB pathway. IKKβ inhibitors, including Bay65-1942, TPCA-1 and PS-1145, have anti-inflammatory effects *in vitro* and *in vivo*, providing strong evidence for the clinical treatment of COPD (Barnes, 2016b). Canakinumab is a monoclonal antibody against IL-1β that specifically binds highly to human IL-1β to inhibit chronic inflammation in COPD, but reliable evidence from human clinical trials is still lacking (Rogliani et al., 2015). MEDI8968 is an all-human monoclonal antibody that selectively binds to IL-1R1 to inhibit IL-1α and IL-1β activation, but in this phase II study, MEDI8968 did not produce significant differences in the incidence of acute exacerbation of COPD in lung function or quality of life improvement (Calverley et al., 2017).

## 4 Summary

Sarcopenia in COPD patients is a complex process involving many pathophysiological factors and molecular pathways. Protein turnover, muscle nucleus turnover and decreased activity, inflammatory response, hypoxia, oxidative stress and drug application play important roles in sarcopenia. Their mechanisms of action cross and influence each other, but the specific molecular mechanism has not been fully elucidated. For COPD with sarcopenia, more clinical trials and basic research are needed to further understand its occurrence and development mechanism, thus providing a theoretical basis for exploring new early screening and treatment methods.
